# Comparison of Nonalbuminuric and Albuminuric Diabetic Kidney Disease Among Patients With Type 2 Diabetes: A Systematic Review and Meta-Analysis

**DOI:** 10.3389/fendo.2022.871272

**Published:** 2022-06-03

**Authors:** Shaomin Shi, Lihua Ni, Ling Gao, Xiaoyan Wu

**Affiliations:** ^1^ Department of Nephrology, Zhongnan Hospital of Wuhan University, Wuhan, China; ^2^ Xiangyang Central Hospital, Affiliated Hospital of Hubei University of Arts and Science, Xiangyang, China

**Keywords:** characteristics, pathology, prognosis, systematic review, nonalbuminuric DKD

## Abstract

**Background:**

Diabetic kidney disease (DKD) is one of most common complications of diabetes. Recently, the classical phenotype of DKD, which is characterized by albuminuria preceding renal insufficiency, has been challenged since a subset of diabetic patients with renal insufficiency but without albuminuria has been increasingly reported. However, the available evidence is inconsistent. Thus, the present systematic review will assess and summarize the available data regarding nonalbuminuric diabetic kidney disease (NADKD).

**Methods:**

PubMed, Embase, and Cochrane were searched for clinical trials related to NADKD. The results were limited to full-text articles published in English, without restrictions on the publication time. The quality of clinical trials was appraised, and the data were extracted. Meta-analysis was conducted using a random-effects model. Descriptive analysis was performed if the data were insufficient.

**Results:**

A final total of 31 articles were included in this review. The meta-analysis of 18 studies showed that compared with albuminuric DKD, patients with NADKD were older (*MD* = 1.04 years old, 95% CI [0.52, 1.57], *p* < 0.05); were more often women (Male *RR* = 0.74, 95% CI [0.68, 0.81], *p* < 0.05); had shorter diabetes duration (*MD* = *−*2.9 years, 95% CI [−3.63, −2.18], *p* < 0.05), lower HbA1c levels (*MD* = *−*0.34%, 95% CI [−0.42, −0.25], *p* < 0.05), and lower blood pressure (systolic blood pressure *MD* = *−*6.21 mmHg, 95% CI [−9.41, −3.0], *p* < 0.05; diastolic blood pressure *MD* = *−*1.27 mmHg, 95% CI [−2.15, 4.0], *p* < 0.05); less frequently experienced diabetic retinopathy (*RR* = 0.58, 95% CI [0.51, 0.67], *p* < 0.05); and less frequently used renin–angiotensin–aldosterone system (RAAS) inhibitors. The underlying pathology of NADKD might be different from that of the classic phenotype of DKD, which is associated with more advanced tubulointerstitial and vascular lesions but mild typical glomerular lesions. The annual estimated glomerular filtration rate decline tended to be lower in patients with NADKD than in those with albuminuric DKD. The risk for cardiovascular disease, end-stage renal disease, and all-cause death was lower for patients with NADKD than patients with albuminuric DKD.

**Conclusions:**

The prevalence of NADKD has increased in recent decades, and its characteristics, pathology, and prognosis are different from those of albuminuric DKD; thus, diagnosis and treatment strategies should be different. More attention should be given to this phenotype.

## Introduction

Diabetic kidney disease (DKD) occurs in approximately 40% of patients with diabetes mellitus (DM) and may result in end-stage renal disease (ESRD), cardiovascular disease (CVD), and even death (1, 2). The classical phenotype of DKD is characterized by the development of persistent albuminuria preceding a progressive decline in glomerular filtration rate (GFR), which is more commonly studied in type 1 diabetes (DM1) (3, 4). However, approximately two decades ago, diminished renal function (GFR < 60 ml/min/1.73 m^2^ was reported to occur without preceding albuminuria in some patients with DM1 (5, 6). Subsequently, this nontypical phenotype of nonalbuminuric diabetic kidney disease (NADKD) has been increasingly recognized, especially in type 2 diabetes (DM2), which is reported to account for approximately 20%–40% of DKD cases (5, 7–13). It is possible that the phenotype of NADKD might follow a different pathophysiological pathway than albuminuric DKD and result in different clinical features and prognoses. Some reports indicated that NADKD might be associated with clinical characteristics such as obesity, aging, female sex, smoking, hypertension, and absence of diabetic retinopathy. However, the pictures described by available reports were inconsistent (14, 15). Regarding prognosis, the risks of ESRD, CVD, and all-cause death were reported to be consistently lower in patients with NADKD than in those with albuminuric DKD. However, inconsistencies exist in the decline rate of the estimated glomerular filtration rate (eGFR) (5, 8). Moreover, few studies have reported mixed findings of pathological changes in NADKD (16–18). Meanwhile, this phenotype is easily overlooked in clinical practice and is often associated with less frequent use of renal protective and cardioprotective treatments. Thus, more attention should be given to NADKD, and we conducted this systematic review to obtain a more comprehensive view of NADKD in patients with type 2 diabetes, which may help to develop more individualized clinical treatment strategies in the future.

## Methods

### Literature Search

PubMed, Embase, and Cochrane were searched systematically for articles relating to NADKD in patients with type 2 diabetes. The search term was “(diabetic nephropathy OR diabetic kidney disease OR diabetic glomerulopathy OR chronic kidney disease in type 2 diabetes OR renal dysfunction in type 2 diabetes OR impaired renal function in type 2 diabetes OR renal impairment in type 2 diabetes OR renal insufficiency in type 2 diabetes OR decline in renal function in type 2 diabetes) AND (non-proteinuria OR non proteinuria OR silent OR without protein OR non-albuminuria OR non albuminuria OR without albuminuria OR normoalbuminuria OR normoproteinuria)”. Limitations were set to retrieve “clinical trial” or “clinical study” (detailed search strategies are shown in the appendix file). The cutoff time for retrieval was November 5, 2021. No time restriction was applied in our search. The results were limited to full-text articles published in English. All of the reference lists from the included studies and relevant reviews were screened manually for additional eligible studies.

### Selection Criteria and Quality Assessment

Two authors independently screened the titles and abstracts for inclusion of all the potential studies identified before. The full text was retrieved if necessary. All clinical studies were included in this evaluation. The literature quality evaluation was conducted by means of the Newcastle–Ottawa Quality Assessment Scale (NOS) for cohort studies and by means of the American Agency for Healthcare Research and Quality (AHRQ) methodology checklist for cross-sectional studies (19). Two authors independently evaluated the results. The NOS included eight items, with a total score of nine, and focused on three areas, including participant selection, comparability of study groups, and ascertainment of exposure. The evaluation tool of the AHRQ included eleven items (19) (details shown in **Appendix Tables 2** and **3**).

### Data Extraction

Data extraction was conducted by the two authors using a standardized data collection form. The following information was obtained from the included studies: type of study, country, first author’s name, year of publication, target population, definition of nonalbuminuria, research features, and all data related to clinical characteristics of patients and follow-up outcomes.

### Statistical Analysis

We systematically analyzed all the parameters of prevalence, clinical characteristics, pathology, and prognosis of patients with NADKD. When parameters were reported by only a few studies, descriptive analysis was performed. However, if the data were sufficient, meta-analysis was performed using RevMan 5.3 and STATA version 16 (Stata Corporation, College Station, TX, USA). The standardized mean difference (SMD) and risk ratio (RR) were calculated using random-effects models for the presence of heterogeneity (*I* 2 > 50%, *p* < 0.05). A *p*-value < 0.05 was considered statistically significant.

## Results

**Figure 1:**
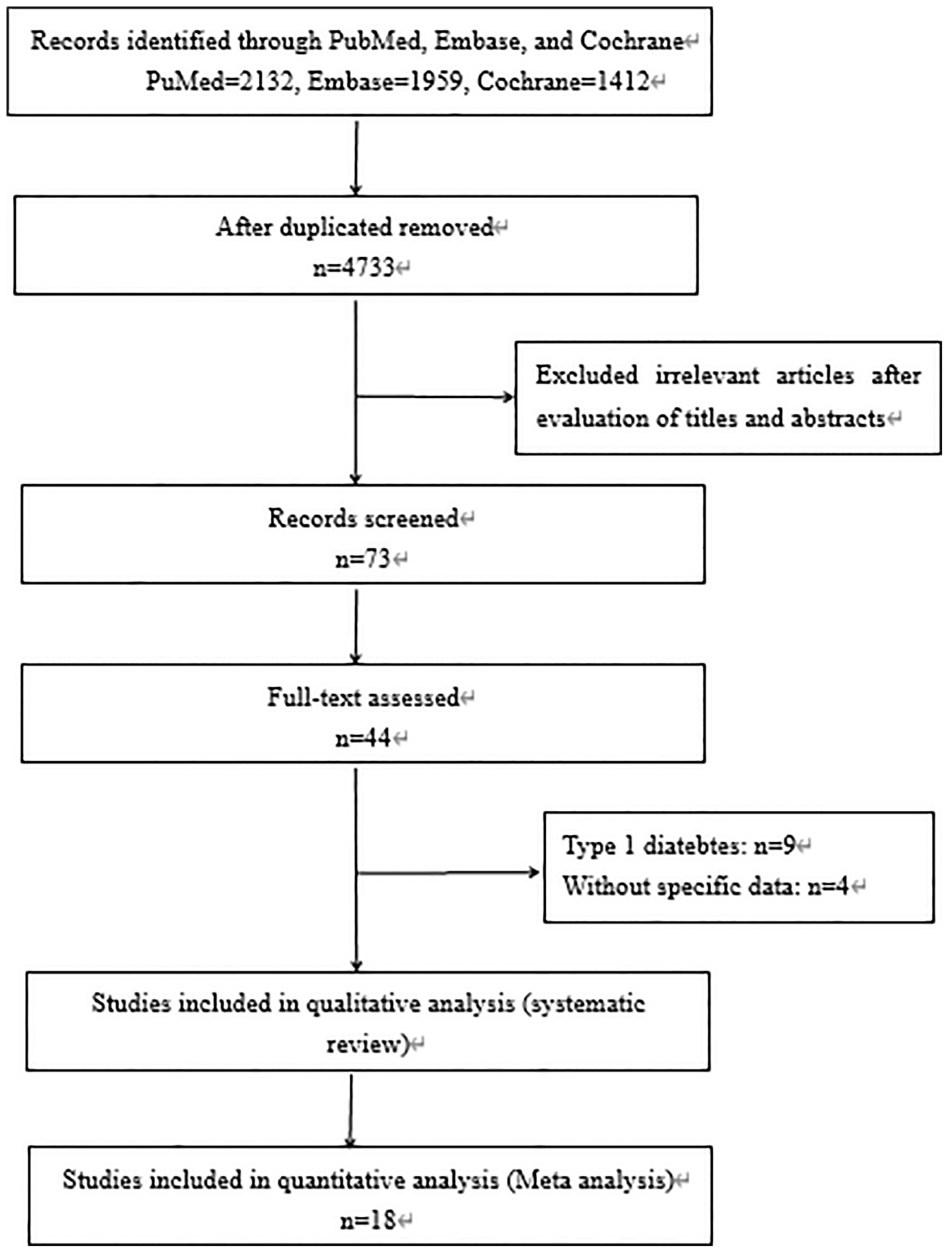
Flow diagram of study selection.

A total of 3,842 papers were identified, of which 44 studies were included by screening the titles and abstracts. Nine full texts associated with type 1 diabetes and 4 studies without specific data were excluded. Finally, 31 available studies regarding the prevalence, characteristics, pathology, or prognosis of NADKD in patients with type 2 diabetes are summarized in **Table 1**, which included 18 cross-sectional, 11 cohort, and 2 case–control studies (flow diagram of study selection shown in **Figure 1**). Participants in these studies came from different regions, including the United States, Italy, Japan, Korea, France, Australia, Denmark, Portugal, Spain, Sweden, and China.

**Table 1:** Available clinical studies related to non-albuminuric diabetic kidney disease in patients with type 2 diabetes.

Study ID	Country	Study type	Patients	NADKD/RI/DKD/DM (number)	Definition of nonalbuminuria	Research features	QE
2003 Holly (20)	US	Cross-sectional	NHANES III	60/171/-/1,197	ACR < 25 mg/g (F) ACR < 17 mg/g(M)	Adults aged ≧40 years old; Highlighted the prevalence	10
2004 Richad (5)	Australia	Cross-sectional	Hospital	43/109/301/-	AER < 20 μg/min	GFR was measured with 99m TC-isotopic estimations	9
2006 Wing (21)	China	Cohort study	Hospital	74/528/-/4,421	ACR < 3.5 mg/mmol	Outcomes; data were collected at the follow-up endpoints	7
2006 Richard (22)	Australia	Cross-sectional	Clinics	39/93/-/325	AER < 20 μg/min	Focus on resistance index of internal arteries	9
2007 Vincent (17)	France	Cohort	Hospital	15/89/-/-	UAE < 30 mg/24 h	Isotopic GFR compared with eGFR; including DM1	8
2007 Caroline (13)	Brazil	Cross-sectional	Clinics	84/-/-/-	AER < 20 μg/min UAE <30 mg/24 h	Compared with normal patients	7
2009 Hiroki (23)	Japan	Cross-sectional	JDDM	262/506/1,261/3,297	ACR < 30 mg/g	–	8
2009 Jee (24)	Korea	Cross-sectional	Clinics	44/151/257/562	ACR < 25 mg/g (F) ACR < 17 mg/g(M)	–	6
2009 Merlin (10)	Australia	Cross-sectional	–	506/920/-/3,893	ACR < 3.5 (F) <2.5 mg/mmol(M)	–	8
2011 Giuseppe (12)	Italy	Cross-sectional	RIACE	1,673/2,959/5,908 15,773	ACR < 30 mg/g	–	8
2011 Rajiv (25)	US	Cross-sectional	Clinic	25/94/-/-	ACR < 220 mg/g	With follow-up data	9
2012 Jamie (11)	Italy	Cross-sectional	DEMAND	1,037/2,586/6,540/11,573	ACR < 30 mg/g	Without specific data	5
2012 Hanri (26)	Sweden	Cross-sectional	NDR	7,337/16,322/-/94,446	AER < 20 μg/min	First analyzed the use of RAS blocker	9
2013 Amy (15)	US	Cross-sectional	NHANES	298/575/1,217/2,798	ACR < 25 mg/g (F) ACR < 17 mg/g(M)	A sample of the US citizens; including DM1	7
2013 Vivek (27)	US	Cross-sectional	Hospital	1,588/-/3,176/15,683	Any one	Focus on racial differences of prevalence	9
2013 Pooja (28)	US	Case–control	Hospital	10/-/-/-	ACR < 30 mg/g	Pathology; collected specimens from tumors	–
2013 Elif (29)	Australia	Case–control	Hospital	8/-/-/-	AER < 20 μg/min	Pathology	–
2013 Mauro (30)	Spain	Cross-sectional	Hospital	17/-/-/-	ACR < 30 mg/g	Composed of patients with GFR < 30 ml/min/m^2^	6
2016 Eunyoung (31)	Korea	Cohort	Clinics	255/1,136/-/-	ACR < 30 mg/g	Reported renal events and CVD	6
2016 Ivo (32)	Portugal	Cross-sectional	Hospital	68/146/-/731	ACR < 30 mg/g	Composed of patients with GFR < 75 ml/min/m^2^	8
2016 Celine (33)	Netherlands	Cross-sectional	Pathology archives	20/-/-/168	UAE < 30 mg/24 h	Pathology; based on autopathy	6
2017 Jong (34)	Korea	Cross-sectional	REBOUND	223/479/1,038/-	ACR < 30 mg/g	Focus on arterial stiffness (baPWV)	6
2018 Digsu (18)	US	Cohort	CRIC	515/1,831/-/-	UAE < 30 mg/24 h	Focus on prognosis; including a small part of DM1	8
2018 Giuseppe (35)	Italy	Cohort	–	–	UAE < 30 mg/24 h	Focus on all-cause mortality	8
2018 Bixia (7)	China	Cohort	Kailuan cohort	940/1,344/2,889/8,811	Urine dipstick -	A large sample size; including DM1	8
2019 Dorte (36)	Denmark	Cohort	clinics	942/1,984/-/-	ACR < 30 mg/g	Focus on eGFR decline	7
2019 Oyunchimeg (37)	Australia	Cohort	ACCORD	432/777/3,644/10,185	ACR < 30 mg/g	Focus on prognosis; large sample size; long follow-up	7
2019 Masayuki (8)	Japan	Cohort	Biopsy registry	88/526/895/-	ACR < 300 mg/g	Based on biopsy	8
2020 Hiroki (16)	Japan	Cohort	Clinics	203/401/1,147/2,953	ACR < 30 mg/g	Focus on prognosis; prior CVD was investigated	7
2020 Hiroyuki (38)	Japan	Cohort	Cohort	96/218/369/-	ACR < 30 mg/g	Focus on prognosis	8
2021 Tsutomu (39)	Japan	Cross-sectional	Regional DM cohort	236/297/342/1,076	ACR < 300 mg/g	Focus on lipidemia; including 5 patients with DM1	6

ID indicates identity; NADKD, nonalbuminuria diabetic kidney disease; IR, renal insufficiency (GFR < 60 ml/min/1.73 m^2^); DKD, diabetic kidney disease; DM, diabetes mellitus; DM1, type 1 diabetes; US, United States; NHANES III, the Third National Health and Nutrition Examination Survey; ACR, albuminuria creatinine ratio; AER, albumin excretion rate; GFR, glomerular filtration rate; UAE, urinary albumin excretion during 24 h; eGFR, estimated glomerular filtration rate; JDDM, Japan Diabetes Clinical Data Management; RIACE, The Renal Insufficiency And Cardiovascular Events; DEMAND, Developing Education on Microalbuminuria for Awareness of Renal and Cardiovascular Risk in Diabetes; RAS, renin–aldosterone system; NDR, Swedish national diabetes register; NHANES, the National Health Nutrition Examination Survey; CVD, cardiovascular disease; REBOUND, a multicenter prospective observational study conducted from December 2008 to December 2010 in Korea; baPWV, brachial-ankle pulse wave velocity; CRIC, chronic renal insufficiency cohort study; ACCORD, Action to Control Cardiovascular Risk in Diabetes clinical trial; QE, literature quality evaluation; -, no record. (Since renal biopsy is not always applicable and rarely performed and three studies based on pathology had small samples, their quality was not evaluated; details are shown in **Appendix Table 2**, **3**.

### Prevalence of Nonalbuminuric Diabetic Kidney Disease

In 1992, Lane et al. identified eight female normoalbuminuric patients with type 1 diabetes who had decreased renal function and diabetic renal structural lesions (40). In 1994, Tsalamandris reported that a subgroup of diabetic patients had renal function decline without significant albuminuria independent of diabetes type (41). This subtype among adults with type 2 diabetes was highlighted in 2003 by Kramer et al. (20). Over the past two decades, the prevalence of albuminuria has decreased; however, the prevalence of eGFR decline has increased, as reported by Kume et al. using 1996, 2001, 2006, and 2004 cohort data from Japanese serial studies conducted at Shiga University of Medical Science (42). NADKD in type 2 diabetes has been increasingly recognized (43–45). In our study, after excluding 5 studies with inconsistent DKD definitions (8, 25, 30, 32, 39) and 2 case–control studies (28, 29), using the available data (missing data were not included), the overall prevalence of NADKD among patients with type 2 diabetes and renal insufficiency was 45.6%; among patients with DKD (either with albuminuria or renal insufficiency), the prevalence of NADKD was 24.7%, and among all the patients with type 2 diabetes, it was 8.4%, which means that almost half of patients with DKD and decreased eGFR did not develop albuminuria.

### Clinical Characteristics of Nonalbuminuric Diabetic Kidney Disease

**Figure 2:**
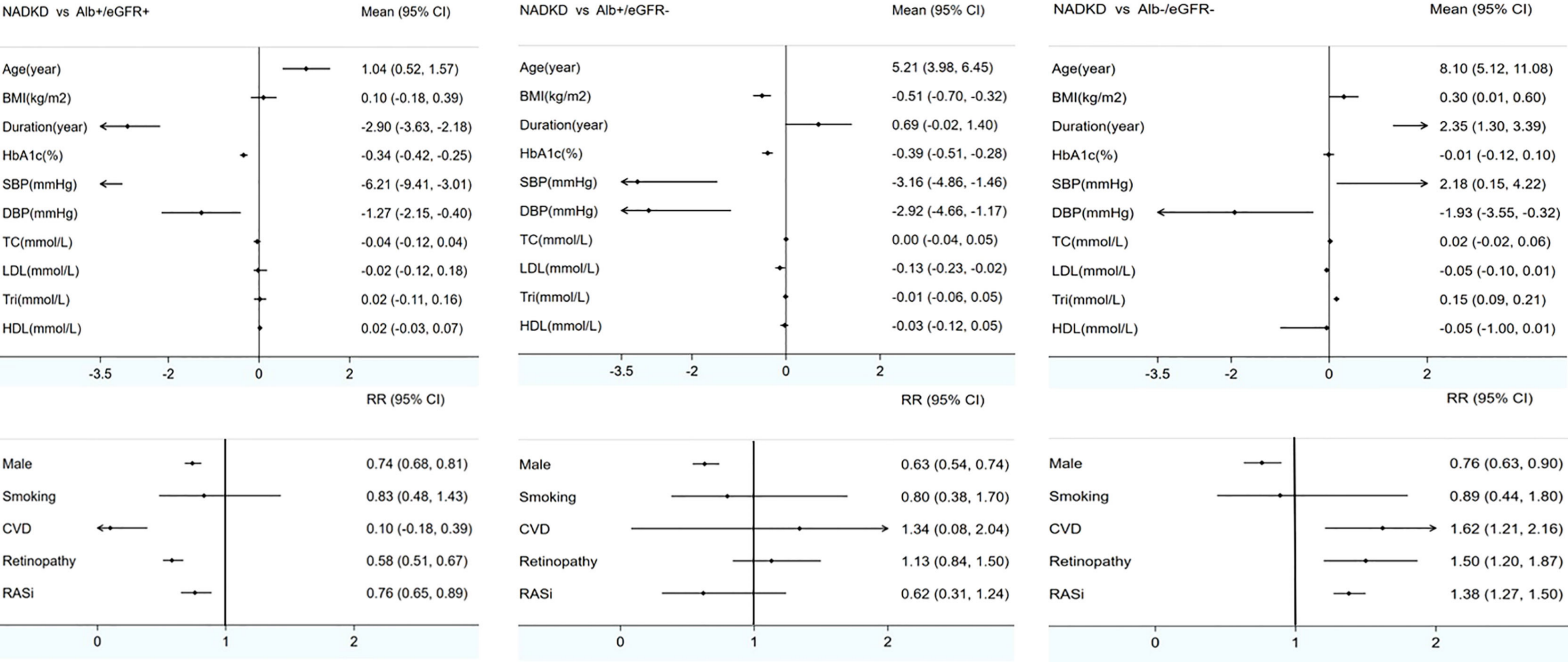
Meta-analyses of clinical characteristics of patients with NADKD compared with patients with Alb^+^/eGFR^-^ and Alb^-^/eGFR^-^ BMI indicates body mass index; HbA1c, glycated hemoglobin; Retino, retinopathy; CVD, cardiovascular disease; SBP, systolic blood pressure; DBP, diastolic blood pressure; TC, total cholesterol; LDL, low-density lipoprotein; Tri, triglyceride; HDL, high-density lipoprotein; RASi, renin–angiotensin system inhibitor; NADKD, normoalbuminuria diabetic kidney disease; Alb+/eGFR+, albuminuria diabetic kidney disease with renal insufficiency; Alb+/eGFR-, albuminuria diabetic kidney disease without renal insufficiency; Alb-/eGFR-, nonalbuminuria diabetic kidney disease without renal insufficiency; Mean, mean difference; RR, risk ratio. Renal insufficiency (GFR < 60 ml/min/1.73 m^2^).

Meta-analysis was used to analyze the clinical characteristics of the patients. Nonalbuminuria was defined as ACR < 17–30 mg/g (albuminuria–creatinine ratio), AER < 20 µg/min (albumin excretion rate), or UAE < 30 mg/24 h (urinary albumin excretion for 24 h). Decreased eGFR was defined as eGFR < 60 ml/min/m^2^. There were 5 studies with inconsistent definitions (2011 Rajiv defined albuminuria as ACR < 220 mg/g; 2019 Masayuki and 2021 Tsutomu defined albuminuria as ACR < 300 mg/g; 2013 Mauro only included patients with GFR < 30 ml/min/m^2^, and 2016 Ivo defined decreased eGFR as <75 ml/min/m^2^ (as shown in **Table 2**) (8, 9, 30, 32, 39). After these 5 studies were excluded, 18 studies reported the characteristics of NADKD, and they were included in the meta-analysis (5, 7, 10, 12, 15–18, 22–24, 26, 31, 34–38) (as shown in Additional **Table 2**). Missing data were not included in the analysis. Compared with patients with albuminuria DKD, patients with NADKD were older (*MD* = 1.04 years old, 95% CI [0.52, 1.57], *p* < 0.05); were more often women (Male *RR* = 0.74, 95% CI [0.68, 0.81], *p* < 0.05); had shorter diabetes duration (*MD* = *−*2.9 years, 95% CI [−3.63, −2.18], *p* < 0.05), lower HbA1c levels (*MD* = *−*0.034%, 95% CI [−0.42, −0.25], *p* < 0.05), and lower blood pressure (systolic blood pressure *MD* = *−*6.21 mmHg, 95% CI [−9.41, −3.0], *p* < 0.05; diastolic blood pressure *MD* = *−*1.27 mmHg, 95% CI [−2.15, 4.0], *p* < 0.05); less frequently experienced diabetic retinopathy (*RR* = 0.58, 95% CI [0.51, 0.67], *p* < 0.05); and less frequently used renin–angiotensin–aldosterone system (RAAS) inhibitors (*RR* = 0.76, 95% CI [0.65, 0.89], *p* < 0.05). However, lipidemia, body mass index (BMI), smoking frequency, and history of CVD were not different between these groups (as shown in **Figure 2**).

**Table 2:** The comparison of eGFR decline between patients with NADKD and albuminuric DKD.

Study ID	Group	N	Follow-up (year)	Annual eGFR decline
2004 Richard	NADKD Alb^+^/eGFR^+^	12 22	3-10	−4.6 ± 1.0 −2.9 ± 0.87
2018 Digsu	NADKD Alb^+^/eGFR^+^	515 1,298	6.3	−0.19 ± 0.11 −3.13 ± 1.69*
2019 Dorte	NADKD Alb^+^/eGFR^+^	942 1,042	3.7	−1.9 ± 0.075 −2.4 ± 0.4*
2019 Oyunchimeg	NADKD Alb^+^/eGFR^+^ Alb^+^/eGFR^-^ Alb^-^/eGFR^-^	423 330 2,814 5,736	8.8	−1.1 ± 1.88 −1.75 ± 2.17 −2.51 ± 2.82 −1.27 ± 2.21
2020 Hiroyuki	NADKD Alb^+^/eGFR^+^ Alb^+^/eGFR^-^ Alb^-^/eGFR^-^	96 122 151 306	4	0 ± 8 (overall change in 4 years) −6 ± 12* (overall change in 4 years) −10 ± 14* (overall change in 4 years) −6 ± 11* (overall change in 4 years)

N indicates number; ID, identifier; eGFR, estimated glomerular filtration rate; NADKD, normoalbumunuria diabetic kidney disease; Alb+/eGFR+, albuminuria diabetic kidney disease with renal insufficiency; Alb+/eGFR-, albuminuria diabetic kidney disease without renal insufficiency; Alb-/eGFR-, nonalbuminuria diabetic kidney disease without renal insufficiency; *, compared with NADKD, p < 0.05.

The comparison between patients with albuminuric DKD without renal insufficiency and patients with NADKD revealed similar results in most respects, but BMI and low-density lipoprotein (LDL) seemed to be lower in the latter, and the frequency of retinopathy and the use of RAAS inhibitors were not different. Finally, when compared with diabetic patients without albuminuria or renal insufficiency, in patients with NADKD, the duration of diabetes was much longer, SBP and triglyceride levels were significantly higher, and the presence of retinopathy, CVD history, and the use of RAAS inhibitors were more frequent (as shown in **Figure 2**; details are shown in **Appendix Table 1** and appendix forest plots).

Heterogeneity test results showed that heterogeneity existed in all the meta-analyses, with *I* 2 fluctuating between 73% and 100% and a *p*-value of less than 0.05. However, further subgroup analyses according to study quality assessment (whether assessed as high quality), study type (cross-sectional or cohort study), study sample (whether the sample of the NADKD group included fewer than 50 cases), and patient sources (whether from hospital sources) showed that the results were robust. No source of heterogeneity was found. Moreover, in sensitivity analysis, when each study was excluded one by one, the results did not change (as shown in Appendix Directory 2).

### Pathology

Since renal biopsy is not always applicable, available data on histological changes in NADKD are limited. Specific renal pathological lesions with diabetes were mostly studied in patients with type 1 diabetes, including thickening of the glomerular basement membrane, mesangial matrix expansion, nodular lesions, and glomerular sclerosis and arteriolar hyalinosis (1). In contrast, this symptom has been less well investigated in patients with type 2 diabetes (DM2). Classic nephropathy lesions in patients with type 2 diabetes are always less severe because the picture of DKD in type 2 diabetes is confounded by other contributors, such as aging, obesity, hypertension, insulin resistance, and vascular disease (1, 46). Few studies have investigated the renal pathological changes in NADKD among patients with DM2; to our knowledge, only four are available. In 2013, Budhiraja et al. collected renal tissue specimens from 10 patients with DM2 and normoproteinuria who underwent nephrectomies because of kidney cancer and first demonstrated that classical diabetic renal pathological changes could occur in the absence of proteinuria (28). Subsequently, the structural lesions of 8 patients with NADKD, 8 with microalbuminuria, and 17 with macroalbuminuria were compared by Ekinci et al., and the results showed that typical histopathological changes in diabetic nephropathy (DN) occurred in patients with DM2 but less frequently than in those with elevated albuminuria (29). In 2016, one study from an autopsy of individuals identified 20 patients with NADKD and demonstrated again that typical DN lesions may develop before the onset of albuminuria and other clinical findings (33). In 2019, Yamanouchi et al. retrospectively assessed 526 patients with type 2 diabetes and DKD [eGFR < 60 ml/min/1.73 m (2)] who had undergone renal biopsy from Japan’s nationwide multicenter renal biopsy registry and showed that normal or near-normal renal structure was the most common (62%) in the patients with NADKD, whereas typical DKD was the most prevalent (66%) in the proteinuric DKD group (8). Overall, NADKD was reported to tend to be associated with more advanced tubulointerstitial and vascular lesions but mild typical glomerular lesions compared with those in patients with albuminuria DKD (7, 29, 47).

### Outcomes of Nonalbuminuric Diabetic Kidney Disease

#### Renal Function Decline

Generally, in the background, the annual decline in eGFR with age in healthy subjects aged 40 years or more is approximately 0.6–1 ml/min/1.73 m^2^, and that in patients with DM but without CKD is approximately 0.4–1.5 ml/min/1.73 m^2^ (5, 37, 38). In the present study, 5 studies focusing on the eGFR decline of patients with NADKD were available. The annual eGFR decline in patients with NADKD was reported to be 0–4.6 ml/min/1.73 m^2^, compared with 1.75–3.13 ml/min/1.73 m^2^ among patients with albuminuric DKD. The annual eGFR decline was slower in NADKD than in albuminuric DKD in most studies except in the 2004 Richardson study (as shown in **Table 3**). The inconsistent results in the 2004 Richardson study may be due to the study design, since it is a cross-sectional study instead of a cohort study, and only 12 out of 43 patients with NADKD included in this study had follow-up data. Moreover, only two studies compared the eGFR decline between patients with NADKD and albuminuric non-DKD or diabetic patients without DKD. Oyuchinmeg Buyadaa et al.´s study in 2019 showed that the annual eGFR decline was lower in patients with NADKD than in patients with albuminuria DKD without renal insufficiency and almost the same when compared with those diabetic patients without DKD (36). However, Hiroyuki Yokoyama et al.’s study showed that the annual eGFR decline in patients with NADKD was even slower than that in diabetic patients without DKD (16).

**Table 3:** Long-term outcomes of patients with type 2 diabetes and non-albuminuric diabetic kidney disease.

Study ID	Group	N	Follow-up (year)	CVD rate (per 1,000 patient-years)	ESRD rate (per 1,000 patient-years)	All-cause mortality rate (per 1,000 patient-years)
2007 Vincent	NADKD Alb^+^/eGFR^+^	15 74	3.2 ± 0.9	–	0 12 (16.2%)* Number of events	0 10 (13.5%)* Number of events
2011 Rajiv	NADKD Alb^+^/eGFR^+^ Alb^-^/eGFR^-^	25 69 35	6.8	66.1 72.5 * 8.6*	46.2 141.9*	105.7 136.8* 50.1*
2016 Eunyoung	NADKD Alb^+^/eGFR^+^	255 881	3.7	41 (16.1%) Number of events 150 (17.0%) Number of events	7 (2.7%) 272 (30.9%)*	
2018 Digsu	NADKD Alb^+^/eGFR^+^	515 1,298	6.3	–	7.4 125.5*	–
2018 Giuseppe	NADKD Alb^+^/eGFR^+^ Alb^+^/eGFR^-^ Alb^-^/eGFR^-^	1,476 1,230 2,966 9,984	7.4 ± 2.1	–	–	30.62 HR 1.58 (1.43, 1.75) 48.65 HR 2.08 (1.88, 2.30) 31.77 HR 1.45 (1.33, 1.58) 18.39 Ref 1
2018 Bixia	NADKD Alb^+^/eGFR^+^ Alb^+^/eGFR^-^ Alb^-^/eGFR	76 50 141 279	6.9	12.3 HR 1.05(0.81-1.35) 19.8 HR 1.48(1.09-2) 14.5 9.5 Ref 1	0.94 HR 31.33 (3.65–269.3) 6.0 HR 267.15 (34.11, 2,092.4) 0.95 0.024 Ref 1*	16.3 HR 1.15 (0.91, 1.44) 38.9 HR 2.7 (2.14, 3.42) 17.2 8.83 Ref 1
2019 Oyunchimeg	NADKD Alb^+^/eGFR^+^ Alb^+^/eGFR^-^ Alb^-^/eGFR^-^	423 345 2,867 2,724	8.8	26.72 HR 1.44 (1.13,1.84) 50.28 HR 2.37 (1.89,2.97) 33.8 HR 1.88 (1.64, 2.16) 14.01 Ref 1.0*	1.81 HR 0.76 (0.34, 1.7) 13.37 HR 4.53 (2.91, 7.01) 5.06 HR 1.72 (1.27, 2.34) 2.6 Ref 1.0	28.14 HR 1.42 (1.14, 1.78) 48.31 HR 2.38 (1.92, 2.9) 30.68 HR 1.82 (1.59, 2.08) 12.58 Ref 1.0*
2019 Masayuki	NADKD Alb^+^/eGFR^+^	88 438	1.9	–	3 (3%) Number of events 101 (23.1%)* Number of events	–
2020 Hiroki	NADKD Alb^+^/eGFR^+^ Alb^+^/eGFR^-^ Alb^-^/eGFR^-^	203 198 746 1,806	9.7 ± 1.1	8.9 HR 1.13 (0.68,1.89) 24.0 HR 2.98 (2.08,4.29) 14.0 HR 2.14 (1.63,2.81) 6.4 Ref 1	4 HR 6.5 (2.46, 17.19) 62.3 HR 105.1(53.4, 204.5) 8.2 HR 13.48(6.85, 26.53) 0.6 Ref 1*	5.1 HR 1.34 (0.68, 2.66) 9.1 HR 2.33 (1.34, 4.07) 4.8 HR 1.65 (1.07, 2.53) 2.9 Ref 1
2020 Hiroyuki	NADKD Alb^+^/eGFR^+^ Alb^+^/eGFR^-^ Alb^-^/eGFR^-^	96 122 151 306	4	7 (7%) Number of events 21 (17%)* Number of events 4 (3%)* Number of events 3 (1%)* Number of events	0 14 (11.5%)* Number of events 0 0	7 (7%) Number of events 16 (13%) Number of events 3 (2%) Number of events 9 (3%) Number of events

N indicates number; ID, identifier; CVD, cardiovascular disease; ESRD, end-stage renal disease; NADKD, normoalbumunuria diabetic kidney disease; Alb+/eGFR+, albuminuria diabetic kidney disease with renal insufficiency; Alb+/eGFR-, albuminuria diabetic kidney disease without renal insufficiency; Alb-/eGFR-, nonalbuminuria diabetic kidney disease without renal insufficiency; Ref, reference; HR, hazard ratio; *, compared with NADKD, p < 0.05; -, no record.

#### Risk of CVD, ESRD, and All-Cause Death

A total of 10 studies reported the risk of CVD, ESRD or all-cause death in patients with NADKD (as shown in **Table 1**). The results were consistent that patients with albuminuric DKD had the highest risk of adverse outcome. However, when comparing between patients with NADKD and patients with albuminuria but without renal insufficiency, the results were conflicting. Three studies showed that patients with NADKD had a lower risk of CVD, ESRD, and all-cause death (7, 35, 37), but Yokoyama et al. reported that the risk of all-cause mortality was higher in patients with NADKD (16). Another recent study showed that patients with NADKD had higher risk of all three adverse outcomes than patients with albuminuric DKD (38). When compared with patients with type 2 diabetes but without DKD, most studies showed that patients with NADKD had higher risk of adverse outcomes, with only one exception (38). In summary, the outcomes of NADKD are better than those of albuminuric DKD, and controversial when compared with diabetic patients without DKD or patients with albuminuria and normal renal function.

## Discussion

### Possible Explanations of Nonalbuminuric Diabetic Kidney Disease

First, the phenomenon may be modified by the development of renoprotective treatments, such as renin–angiotensin system inhibitors (RASi), lipid-lowering drugs, and sodium glucose cotransporter 2 inhibitors (SGLT2i), which reduce urinary albuminuria. However, RASi was not an independent risk factor for NADKD in multivariate analysis (26), and when excluding users of RASi or lipid-lowering drugs, the prevalence and general characteristics of NADKD resembled those of all patients (12, 23, 39, 48). Second, NADKD may be an early stage of classic DKD. As shown in the present systematic review, NADKD was associated with a shorter duration of diabetes and a lower prevalence of retinopathy. Moreover, in An et al.´s study, the prevalence of normal albuminuria decreased progressively with an increase in the diabetic duration, and among the 30 diabetic patients with renal insufficiency who had a diabetic duration longer than 21 years, none had normal albuminuria (24). Thus, it is possible that at least a proportion of patients with NADKD may be at an early stage of DKD. Third, the prevailing underlying pathology of patients with NADKD might be macroangiopathy instead of microangiopathy. In the present study, patients with NADKD were older than other diabetic patients, less frequently had concurrent diabetic retinopathy, and tended to have lower HbA1c levels and LDL and lower blood pressure. On the other hand, the annual eGFR decline tended to be slower in patients with NADKD and was even slower than in patients with nonalbuminuria and non-DKD in Yokoyama et al.’s study (16). However, the risk of CVD and all-cause mortality was higher than that in patients with nonalbuminuria and non-DKD and tended to be comparable to that in patients with albuminuria and normal renal function. These findings suggest that renal impairment in NADKD was not mainly caused by hyperglycemia or microangiopathy, and it is possible that aging, arteriosclerosis, and macroangiopathy might contribute more to NADKD. In accordance, Kume et al. reported that eGFR decline without albuminuria was associated with age-dependent arterial stiffness (42). Kim et al. demonstrated that arterial stiffness (measured by baPWV) was strongly related to albuminuria in patients with type 2 diabetes (34). However, MacIsaac et al. reported that the resistance index of the intrarenal arteries measured by duplex Doppler ultrasound, which reflects intrarenal arteriosclerosis elevation, was not different between patients with NADKD and those with albuminuric DKD (22). Fourth, genetic susceptibility might also play a role in the development of NADKD. Bhalla et al. reported that non-Hispanic white individuals are more likely to have nonproteinuric DKD than individuals of other races (27). A Japanese study reported that DKD in the absence of albuminuria was associated with polymorphisms of the protein kinase C-β gene (49). Yagil et al. identified an experimental model reminiscent of human NADKD and reported that the absence of proteinuria in Cohen diabetic sensitive rats with declined renal function may be genetically determined (50). Fifth, the divergence of pathologic changes may have played a role. The different clinical characteristics of NADKD mean that the underlying renal pathology may be different. The pathological characteristics of NADKD were different from those of albuminuria DKD. Klessens et al. reported that the presence of albuminuria was correlated with interstitial fibrosis tubular atrophy (IFTA) rather than DN, and it is possible that the onset of albuminuria may only occur after interstitial and tubular damages are severe enough to lead to insufficient reabsorption of the leaked protein from the glomerular filtrate (33). As a result, renal insufficiency due to glomerular damage may occur preceding the onset of albuminuria. Finally, the preponderance of the disease in female patients indicated that the pathogenesis of NADKD might be affected by estrogen. NADKD may be caused by multipathogenic factors. Superimposed factors such as cholesterol emboli, hypertensive nephrosclerosis, episodes of acute kidney injury, and concomitant additional renal disease have all been suggested to explain this situation.

### Trajectories of Nonalbuminuric Diabetic Kidney Disease

Generally, DKD is considered to initiate with an increase in urinary albumin excretion, then progress to macroalbuminuria, and finally develop to renal insufficiency (16). However, this process has been challenged since trajectories different from this classical phenotype have been increasingly reported. In recent decades, the phenotype of NADKD has been increasingly recognized (14). Patients with NADKD tend to have a lower eGFR decline rate, which is even slower than that in diabetic patients without DKD (16), and they have a lower risk of adverse end-stage outcomes than patients with albuminuric DKD. The situation of this phenotype was relatively stable compared with those of other phenotypes. However, these patients still have a higher risk of ESRD, CVD, or death than diabetic patients without DKD, which indicates that a subset of patients might progress gradually to the end stage without albuminuria. In addition, it is possible that some patients might experience a rapid decline in eGFR (defined as ≥5 ml/min/1.73 m^2^/year), and the reported risk factors include high GFR, high systolic blood pressure, prior CVD, and albuminuria ^2^. Krolewski et al. observed the development of 286 patients with type 1 diabetes and normoalbuminuria for 4–10 years and showed that 10% of them experienced a rapid eGFR decline (at least 3.3%/year) preceding the onset of albuminuria. Of note, most patients in his study had normal renal function (51). Thus, although a rapid eGFR decline was common in diabetic patients with albuminuria, the development of albuminuria was not indispensable for it. Alternatively, some patients with NADKD may develop albuminuric DKD, as we discussed above.

### Outlook of Nonalbuminuric Kidney Disease

Over the past two decades, among diabetic patients, although the prevalence of albuminuria has decreased, the prevalence of eGFR <60 ml/min/1.73 m^2^ is increasing (16), which means that the natural history of DKD may have changed worldwide. NADKD has already become the predominant phenotype of DKD with renal insufficiency, and its prevalence may increase in the future.

Although studies associated with NADKD have increased in recent decades, the understanding of NADKD is still far from sufficient. Microalbuminuria is always considered the first clinical sign of NADKD. However, the discordance between changes in albuminuria and GFR indicated that albuminuria cannot meet the diagnostic demand for early DKD in clinical practice. Available studies have reported the use of some new markers, ultrasound technology (such as the renal-resistive index), or other methods in the early diagnosis of DKD among patients with nonalbuminuria and normal renal function (52, 53). Cytokines and growth factors (IL-17A and macrophage inflammatory protein 1α) related to mild inflammation and fibrosis were reported to be associated with NADKD (54). However, no other studies have specifically examined the diagnosis of NADKD. Moreover, since NADKD is different from albuminuria DKD, the therapeutic strategies should be different. During the past two decades, renoprotective drugs have mostly aimed at reducing albuminuria. The prevalence of albuminuria in patients with DKD decreased over the past decade, which indicated that the treatment was effective (42). However, patients with NADKD are usually excluded from relevant clinical trials; thus, whether these strategies are sufficient to combat NADKD is unknown. At present, there is no specific treatment for NADKD. Therefore, further studies are needed to provide an additional understanding of NADKD.

### Strengths and Limitations

To our knowledge, our study is the first systematic review associated with NADKD. Thirty-one available studies from different countries were included for analysis, among which 20 studies were evaluated as high quality and 9 were evaluated as medium quality. However, there are some limitations. First, most studies are not based on biopsy for the diagnosis of DKD and thus may include some other kidney diseases. However, studies based on biopsy showed that the incidence of nondiabetic nephropathy was relatively low (29, 33). Second, most studies used eGFR, which might not accurately reflect the real GFR. However, it has been reported that the difference between the isotopic determination of GFR and the MDRD equation estimation of GFR is slight and not significant (17). Third, patients in most studies had only one assessment of albuminuria without confirmation. Fourth, patient characteristics, laboratory methods, and study designs were different among the included studies. Fifth, there might be divergences among different races and countries, but we cannot determine them. Thus, our results should be interpreted with caution due to the above limitations and the heterogeneity in the meta-analysis.

## Conclusion

Patients with NADKD were older, were more often women, had shorter diabetes duration and lower HbA1c levels and lower blood pressure, and were more frequently absent of diabetic retinopathy and less frequently used RAAS inhibitors than patients with albuminuric DKD. The underlying pathology of NADKD is different from the classic phenotype of DKD. Patients with NADKD tended to have a relatively better prognosis than those with albuminuric DKD. The prevalence of NADKD has increased in recent decades, but studies associated with the etiology, clinical features, trajectories, diagnosis, interventions, or prognosis of NADKD are insufficient and urgently needed. More attention should be given to this phenotype, which is easily overlooked, and further studies and a deeper understanding of NADKD would enable more individualized therapy for these patients, which might ultimately mitigate disease progression and the burden of patients with NADKD in the future.

## Data Availability Statement

The original contributions presented in the study are included in the article/**Supplementary Material**. Further inquiries can be directed to the corresponding authors.

## Author Contributions

SS and LN designed the study and participated in data collection. SS and LN performed the meta-analysis and drafted the manuscript. LG and XW partially conceived the research idea and edited the manuscript. All authors contributed to the article and approved the submitted version.

## Funding

This work was supported by grants from the Fundamental Research Funds for Central University (2042020kf0137); the Zhongnan Hospital of Wuhan University Science, Technology and Innovation Seed Fund, Project znpy2019036 and znpy2017044; the Hubei Province Health and Family Planning Scientific Research Project (WJ2019MB103); the Clinical Research Project for Wu Jieping Medical Foundation (320.6750.19089-58); and the Research Fund from Medical Sci-Tech Innovation Platform of Zhongnan Hospital, Wuhan University (PTXM2020028).

## Conflict of Interest

The authors declare that the research was conducted in the absence of any commercial or financial relationships that could be construed as a potential conflict of interest.

## Publisher’s Note

All claims expressed in this article are solely those of the authors and do not necessarily represent those of their affiliated organizations, or those of the publisher, the editors and the reviewers. Any product that may be evaluated in this article, or claim that may be made by its manufacturer, is not guaranteed or endorsed by the publisher.
