# Supplementary material for: Comparison of Nonalbuminuric and Albuminuric Diabetic Kidney Disease Among Patients With Type 2 Diabetes: A Systematic Review and Meta-Analysis
Source: Front Endocrinol (Lausanne). 2022 Jun 3;13:871272. doi: 10.3389/fendo.2022.871272 (PMC9203723; doi:10.3389/fendo.2022.871272)
Supplement: Supplementary file 1 [file DataSheet_1.docx]

Appendix Table 1 Clinical characteristics of the patients in studies included in the meta-analysis

| Study ID | Number | Age  (year) | Male(%) | Duration  (year) | BMI  (kg/m^2^) | RO  % | CVD % | Sm  % | HbA1c  Levels  (%) | SBP  DBP  (mmHg) | Lipidemia  Cholesterol, LDL, Triglyceride, HDL  (mmol/L) | RASi% | AER (ug/min)  UAE (mg/24h)  ACR (mg/g) | GFR  ml/min/m^2^ |
| --- | --- | --- | --- | --- | --- | --- | --- | --- | --- | --- | --- | --- | --- | --- |
| 2004  Richard | NADKD 43  Alb^+^ /eGFR^+^ 66 | 73±1  70±3.2 | 44  66.5 | 14±1.0  15.6±1.6 | 30.8±1.0  30.3±1.6 | 26  46 | - | 38  35 | 7.3±0.3  7.9±0.25 | 138±3;75±2,  147±3;78±1 | 4.4±0.2; 2.6±0.1; 1.9±1.1; 1.15±0.05  4.4±0.2; 2.7±0.2; 1.9±1.1; 1.1±0.12 | 74  51 | - | 47±2  44±4.5 |
| 2006  Richard | NADKD 39  Alb^+^ /eGFR^+^ 54 | 72±1  70±3.6 | 51  72 | 17±2  15±2 | 31.8±0.9  31±0.9 | - | - | - | 7.2±0.2  7.8±0.35 | 143±3;77±2,  147±3;78±2 | 4.3±0.1; 2.5±0.1; 1.6±0.2; 1.15±0.05  4.5±0.1; 2.6±0.1; 1.5±0.3; 1.12±0.7 | 74  51 | 9±1.5ug/min  604±651ug/min | 45±2  41±6.1 |
| 2007  Vincent | NADKD 15  Alb^+^ /eGFR^+^ 74 | 68±9  64±12 | 36  56 | 14±5  19±11 | 27.0±4.5  26.9±4.3 | 26  66 | - | 2052 | 9.0±1.3  8.5±1.6 | 143±16; 79±8  147±19;81±10 | - | 60  75 | 20.7±6.2mg/24h  712±876mg/24h | 47.5±19.9  45.2±31.4 |
| 2009  Hiroki | NADKD 262  Alb^+^ /eGFR^+^ 244  Alb^+^ /eGFR^-^ 755  Alb^-^ /eGFR^-^  2036 | 62±6  62±6  58±8  57±9 | 52.7  64.7  65.5  64.6 | 11±8  15.7±9  12±7  10±7 | - | 23  73  44  23 | 13  25  11  6.4 | 41  53  56  52 | 6.8±0.9  7.1±1.1  7.4±1.2  7.0±1.0 | 130±16; 75±10  135±16;76±10  131±14; 74±9  127±14;74±9 | 5.2±0.78; - ;1.46±0.27; 1.4±0.36  5.3±1.18; - ;1.64±0.31; 1.3±0.46  5.24±1.0; - ;1.45±0.31; 1.4±0.47  5.1±0.83; - ;1.3±0.25; 1.42±0.41 | - | - | - |
| 2009  Jee | NADKD 44  Alb^+^ /eGFR^+^ 107  Alb^-^ /eGFR^-^ 305 | 59±11  59±9  51±7 | 23  51  53 | 5.5±2.3 15.0±7  5.7±2.8 | 25±2.6  24±2.8  24±3.1 | 15  69  11 | 27  43  12 | 1819  13 | 7.0±1.1  7.4±1.2  7.2±1.4 | 127±16;72±11  133±16; 76±10  119±18;75±12 | 4.4±1.03; 2.5±0.83;1.7±0.94;1.2±0.31  4.5±0.95;2.5±0.77;1.8±0.95;1.15±0.27  4.8±1.06;2.7±0.96;1.7±1.09;1.30±0.28 | 4762  18 | 8.5±4.8mg/g  1608±1458mg/g  8.5±5.1mg/g | 48.3±5.2  31.3±14.5  75.3±9.8 |
| 2009  Merlin | NADKD 506  Alb^+^ /eGFR^+^ 414 | 73±1  73±1.7 | 36  52 | 9±1  11.3±1 | - | 1019 | 41 53 | 4337 | 7 ±0.1  7.4±0.16 | 135±1;75±1  135±2 ;75±1 | - ;2.4±0.1;1.9±0.1;1.2±0.1  - ;2.2±0.1;2.1±0.1; 1.3±0.1 | 8085 | - | - |
| 2011  Giuseppe | NADKD 1673  Alb^+^ /eGFR^+^  1286  Alb^+^ /eGFR^-^  2949  Alb^-^ /eGFR^-^  9865 | 73±3  73±3  66±4  58±2 | 34.4  60.1  70.7  56.0 | 14±4  18±4  12±4  9±4 | - | 34  42  30  17 | 32  45  27  18 | 34  47  51  42 | 7.4±0.2  7.6±0.25  7.6±0.25  7.2±0.15 | 140±6; 80±3  140±5;80±3  140±5;80±4  135±6;80±4 | 4.74±0.3;2.7±0.28; 1.46±0.36; -  4.7±0.35;2.6±0.29; 1.61±0.28; -  4.7±0.34;2.67±0.3; 1.42±0.25; -  4.7±0.31; 2.77±0.3;1.27±0.21; - | 69  77  68  51 | 10.2±4mg/24h  125±82mg/24h  70±25.7mg/24h  9.3±2.5mg/24h | 52.1±3  46.6±4.3  82.5±6.5  83.7±5.9 |
| 2012  Hanri | NADKD 10111  Alb^+^ /eGFR^+^ 6211  Alb^+^ /eGFR^-^ 13347  Alb^-^ /eGFR^-^ 51646 | 76±8.7  76±8.8  68±11.1  66±11 | 36  58  68  57 | 10.1±7.7  13.0±8.2  9.5±7.1  7.5±6.4 | 29.3±5.2  29.5±5.3  30.0±5.4  29.5±5.3 | 20  31  22  16 | 31  39  23  17 | 6.1  8  16  13 | 7.0±3.2  7.2±3.4  7.2±3.4  6.9±3.2 | 136±17; 73±9.8  139±19;74±10.3  139±17; 77±9.9  136±16;77±9.3 | 4.8±1.1; 2.7±0.9 ;1.9±1.0; 1.3±0.4  4.7±1.1; 2.6±0.9 ;2.1±1.2; 1.2±0.4  4.8±1.1; 2.7±0.9 ;1.9±1.2; 1.2±0.4  4.8±1.0; 2.8±0.9;1.7±1.0; 1.3±0.4 | 73  78  -  - | - | 49±8.6  44±74.1  88±21.2  88±18.9 |
| 2013  Amy | NADKD 298  Alb^+^ /eGFR^+^ 277 | 71±1  72±1 | 42  48 | - | - | - | 38 56 | - | - | 133±2;61±4  146±2;65±2 | - ; - ;2.82±0.1;1.32±0.03  - ; - ;2.54±0.1;1.27±0.03 | 4453 |  | 49±0.5  42±1 |
| 2016  Eunyoung | NADKD 255  Alb^+^ /eGFR^+^ 881 | 65±8.3  61±11.3 | 55.7  68.6 | 9.9±8.4  12.4±8.8 | 24.5±3.2  24.7±8.7 | - | 35  29 | - | 7.5±1.5  7.8±1.7 | - | 4.4±1.1; 2.65±0.9; 2.1±01.4; 1.14±0.28  4.5±1.45; 2.73±1.02; 2.0±1.4; 1.1±0.37 | - | 17.4±7.8mg/g  1145±1689mg/g | 45.3±11.8  37.2±14.1 |
| 2017  Jong | NADKD 223  Alb^+^ /eGFR^+^  256  Alb^+^ /eGFR^-^  559  Alb^-^ /eGFR^-^  1575 | 67±9  65±10  59±11  58±10 | 26  40.7  51.2  43.5 | 10±7  13±8  10±8  8±7 | 25.2±3  25±3.4  25.4±3.9  24.7±3.2 | - | - | 8.7  16  27  22 | 7.5±1.5  7.8±1.8  8.1±1.8  7.4±1.5 | 131±18;76±10  140±22;78±12  132±17;80±10  126±14;78±9 | - ;2.4±0.83; 1.2±0.31; 1.3±0.23  - ;2.4±0.93; 1.14±0.3; 1.5±0.24  - ;2.5±0. 80; 1.24±0.3; 1.47±0.3  - ;2.4±0.86; 1.27±0.3; 1.2±0.23 | 69  77  68  51 | 10.2±4mg/24h  125±82mg/24h  70±25.7mg/24h  9.3±2.5mg/24h | 52.1±3  46.6±4.3  82.5±6.5  83.7±5.9 |
| 2018  Digsu | NADKD 515  Alb^+^ /eGFR^+^  1298 | 61±9  59±10 | 41.2  62.1 | - | - | - | - | 9.7  13 | 7.2±1.4  7.8±1.7 | 122±18;65±11,  138±22;72±13 | - | 83  78 | - | 47.9±16.2  38.5±13.2 |
| 2018  Giuseppe | NADKD 1476  Alb^+^ /eGFR^+^  1230  Alb^+^ /eGFR^-^  2966  Alb^-^ /eGFR^-^  9984 | 74±8  73±9  66±10  65±10 | 39.8  62.3  70.0  54.8 | 16.4±11  18.3±11  13.8±10  11.9±9.6 | 29.1±5.1  29.1±5.1  29.6±5.3  28.7±5.1 | 25  48  30  17 | 34  46  51  42 | 37  47  27  22 | 7.6±1.45  7.8±1.65  7.9±1.68  7.4±1.41 | 139±19;77±10  141±20;78±10  140±19;80±10  137±17;79±9 | 4.79±1; 2.77±0.9; 1.5±0.25; 1.27±0.36  4.78±1; 2.73±0.9; 1.6±0.27; 1.20±0.36  4.76±1; 2.75±0.9; 1.4±0.25; 1.25±0.35  4.79±1; 2.81±0.8; 1.3±0.21; 1.32±0.35 | 73  78  69  53 | 10.5±2.9mg/24h  126±86mg/24h  70±26.0mg/24h  9.3±2.5mg/24h | 48.5±9.5  43.0±12.3  86.3±15.2  87.8±14.1 |
| 2018Bixia | NADKD 940  Alb^+^ /eGFR^+^  404  Alb^+^ /eGFR^-^  1545  Alb^-^ /eGFR^-^  5922 | 62±10  62±10  56±11  55±10 | 72  79  89  84 | 10±7  13±8  10±8  8±7 | 25.9±3.4  26.4±3.6  26.4±3.7  26.0±3.3 | - | - | 31  29  46  45 | - | 141±21;85±11  146±23;88±12  145±22;90±13  138±20;87±11 | 5.2±2.0; 2.8±1.0; 2.0±1.8; 1.3±0.4  5.2±1.1; 2.8±0.9; 2.0±1.4; 1.4±0.3  5.5±1.7; 2.9±1.1; 2.4±2.7; 1. 5±0.4  5.3±1.4; 2.7±1.4; 2.1±2.6; 1.5±0. 5 | - | Urine stick test | 51.8±7.5  49.3±9.2  85.9±16  88.7±16.1 |
| 2019Dorte | NADKD 942  Alb^+^ /eGFR^+^  1042 | 71±8.5  68±9.9 | 47.7  66.4 | 12.8±2.8  13.9±2.9 | 30.4±5.6  30.1±5.7 | 54  69 | - | 65  57 | 8.1±1.6  8.4±1.7 | 137±21;74±11,  145±23;67±17 | 4.6±1.2; 2.4±1.0; 1.8±0.35; 1.3±0.4  4.8±1.3; 2.5±1.1; 2.1±0.4; 1.2±0.40 | 52  58 | 9±3mg/g  338±384mg/g | 53.5±3  51.3±3.8 |
| 2019  Oyunchimeg | NADKD 432  Alb^+^ /eGFR^+^  345  Alb^+^ /eGFR^-^  2867 | 67±2.4  66±2.3  62±2.4 | 41.6  49.8  68.5 | 10±2.5  15±3.3  10±2.8 | 32.4±5.4  32.7±5.7  32.4±5.5 | 17  30  16 | 40  48  41 | 6  13  17 | 8.1±1.0  8.3±1.0  8.4±1.1 | 133±17;71±11  145±19;73±12  142±18;76±11 | 4.8±1.1; - ; 1.9±0.35; 1.1±0.3  4.8±1.2; - ; 2.1±0.38; 1.0±0.3  4.8±1.2; - ; 1.8±0.37; 1.1±0.3 | 78  78  74 | 10.0±3mg/g  128±95.3mg/g  90±46mg/g | 53.7±2.4  53.1±2.4  90.3±7.2 |
|  | Alb^-^ /eGFR^-^ 6541 | 62±3.0 | 60.2 | 8.5±2.4 | 32.0±5.3 | 7.5 | 32 | 13 | 8.2±1.0 | 134±16;76±10 | 4.7±1.0; - ; 1.7±0.33; 1.1±0.3 | 66 | 8.2±2.3mg/g | 94.5±18.2 |
| 2020  Hiroki | NADKD 203  Alb^+^ /eGFR^+^  198  Alb^+^ /eGFR^-^  746  Alb^-^ /eGFR^-^  1806 | 63±5.8  62±6.2  58±8.4  58±8.4 | 57.1  63.6  76.0  63.5 | 11.7±8.2  13.8±8.4  11.5±7.4  10.5±7.6 | 25.0±4.1  25.2±4.0  25.8±4.2  24.2±3.4 | - | 14  26  12  7.4 | 42  54  56  51 | 7.2±0.89  7.4±1.11  7.8±1.23  7.3±0.97 | 128±13;75±9.2  133±16;75±11  132±13;76±8.8  127±14;74.1±9 | - ; - ; - ; 1.39±0.4  - ; - ; - ; 1.37±0.4  - ; - ; - ; 1.39±0.4  - ; - ; - ; 1.44±0.4 | 3451  31  20 | 10.0±2.2mg/g  188±234mg/g  87±55.8mg/g  9.9±2.4mg/g | 53.7±5.4  49.4±7.8  84.1±16.4  81.8±14.3 |
| 2020  Hiroyuki | NADKD 96  Alb^+^ /eGFR^+^  122  Alb^+^ /eGFR^-^  151  Alb^-^ /eGFR^-^  306 | 71±10  72±9  64±13  62±11 | 52  64  64  55 | 14±9  16±11  12±10  10±9 | 25.7±4.4  25.6±4.3  26.1±5.3  25.4±4.2 | 28  53  34  21 | 53  65  34  26 | 27  43  39  33 | 7.1±1.0  7.5±1.3  7.7±1.5  7.4±1.3 | 127±17;72±13  131±16;72±13  134±15;78±12  130±15;76±11 | - ; 2.23±0.6; - ; 1.29±0.34  - ; 2.33±0.8; - ; 1.28±0.40  - ; 2.52±0.75; - ; 1.39±0.36  - ; 2.46±0.75; - ; 1.4±0.39 | - | - | 49±10  44±13  83±19  82±15 |

Abbreviations: BMI indicates body mass index; ID, identity; RO, retinopathy; CVD, cardiovascular disease; Sm, smoking; HbA1c, glycated hemoglobin; SBP, systolic blood pressure; DBP, diastolic blood pressure; LDL, low density lipoprotein; HDL, high density lipoprotein; RASi, renin angiotensin system inhibitor; AER, albumin excretion rate; UAE, urinary albumin excretion; ACR, albumin creatinin ration; GFR, glomerular filtration rate; NADKD, normoalbumunuria diabetic kidney disease; Alb+ /eGFR+, albumimuria diabetic kidney disease with renal insufficiency; Alb+ /eGFR-, albumimuria diabetic kidney disease without renal insufficiency; Alb- /eGFR-, nonalbumimuria diabetic kidney disease without renal insufficiency; -, no record.

Appendix Table 2 Assessment of the risk of bias in cross-sectional studies

| Study ID | 1 | 2 | 3 | 4 | 5 | 6 | 7 | 8 | 9 | 10 | 11 | Total  Score |
| --- | --- | --- | --- | --- | --- | --- | --- | --- | --- | --- | --- | --- |
| 2003Holly |  |  |  |  |  |  |  |  |  |  | -1 | 10 |
| 2004Richard |  |  |  | -1 |  |  |  |  | -1 |  |  | 9 |
| 2006Richard |  |  |  |  |  |  |  |  |  | -1 | -1 | 9 |
| 2007Caroline |  |  |  |  |  |  | -1 | -1 | -1 |  | -1 | 7 |
| 2009Hiroki |  |  |  |  |  |  | -1 |  | -1 |  | -1 | 8 |
| 2009Jee |  |  |  |  |  | -1 |  | -1 | -1 | -1 | -1 | 6 |
| 2009Merlin |  |  |  |  |  | -1 |  |  | -1 |  | -1 | 8 |
| 2011Giuseppe |  |  |  |  |  |  |  |  | -1 | -1 | -1 | 8 |
| 2011Rajiv |  |  |  |  |  |  |  | -1 | -1 |  |  | 9 |
| 2012Jamie |  |  |  |  |  | -1 | -1 | -1 | -1 | -1 | -1 | 5 |
| 2012Hanri |  |  |  |  |  |  |  |  | -1 |  | -1 | 9 |
| 2013Amy |  |  |  |  |  |  | -1 |  | -1 | -1 | -1 | 7 |
| 2013Vivek |  |  |  |  |  |  |  |  |  | -1 | -1 | 9 |
| 2013Mauro |  |  |  | -1 |  | -1 |  |  | -1 | -1 | -1 | 6 |
| 2016Ivo |  |  |  |  |  |  |  |  | -1 | -1 | -1 | 8 |
| 2016Celine |  |  |  | -1 |  | -1 |  | -1 |  | -1 | -1 | 6 |
| 2017Jong |  |  |  |  |  | -1 |  | -1 | -1 | -1 | -1 | 6 |
| 2021Tsutomu |  |  |  |  |  | -1 |  | -1 | -1 | -1 | -1 | 6 |

Abbreviation: ID indicates identity. ARHQ (The Agency for Healthcare Research and Quality) methodology checklist was used. 11 items are included, and each item use "Yes (1 point)", "Unclear or No (0 point)" to judge. The total score is 11, 0~3 is low quality, 4~7 is medium quality, 8~11 is high quality. Items: 1.Define the source of information (survey, record review); 2.List inclusion and exclusion criteria for exposed and unexposed subjects (cases and controls) or refer to previous publications; 3.Indicate time period used for identifying patients; 4.Indicate whether or not subjects were consecutive if not population-based; 5.Indicate if evaluators of subjective components of study were masked to other aspects of the status of the participants ; 6.Describe any assessments undertaken for quality assurance purposes (e.g., test/retest of primary outcome measurements); 7.Explain any patient exclusions from analysis; 8.Describe how confounding was assessed and/or controlled; 9.If applicable, explain how missing data were handled in the analysis ; 10.Summarize patient response rates and completeness of data collection; 11.Clarify what follow-up, if any, was expected and the percentage of patients for which incomplete data or follow-up was obtained.

Appendix Table 3 Assessment of the risk of bias in cohort studies

| Study ID | 1 | 2 | 3 | 4 | 5 | 6 | 7 | 8 | Total  Score |
| --- | --- | --- | --- | --- | --- | --- | --- | --- | --- |
| 2006Wing |  |  |  |  | -2 |  |  |  | 7 |
| 2007Vincent |  |  |  |  | -1 |  |  |  | 8 |
| 2016Eunyoung |  |  |  |  | -2 |  |  | -1 | 6 |
| 2018Digsu |  |  |  |  | -1 |  |  |  | 8 |
| 2018Giuseppe |  |  |  |  | -1 |  |  |  | 8 |
| 2018Bixia |  |  |  |  | -1 |  |  |  | 8 |
| 2019Dorte |  |  |  |  | -1 |  |  | -1 | 7 |
| 2019Oyunchimeg |  |  |  |  | -1 |  |  | -1 | 7 |
| 2019Masayuki |  |  |  |  | -1 |  |  |  | 8 |
| 2020Hiroki |  |  |  |  | -1 |  |  | -1 | 7 |
| 2020Hiroyuk |  |  |  |  | -1 |  |  |  | 8 |

Abbreviation: ID indicates identity. NOS (Newcastle-Ottawa Scale) was used. 8 items are included, with 1 point for each item, but item 5 was given 2 points to judge. The total score is 9, 0~3 is low quality, 4~6 is medium quality, 7~9 is high quality. Items: Selection: 1.Representativeness of the exposed cohort; 2.Selection of the non exposed cohort; 3.Ascertainment of exposure; 4.Demonstration that outcome of interest was not present at start of study; Comparability: 5.Comparability of cohorts on the basis of the design or analysis; Outcome: 6.Assessment of outcome; 7.Was follow-up long enough for outcomes to occur;8.Adequacy of follow up of cohorts.
