# Supplementary material for: Comparison of Nonalbuminuric and Albuminuric Diabetic Kidney Disease Among Patients With Type 2 Diabetes: A Systematic Review and Meta-Analysis
Source: Front Endocrinol (Lausanne). 2022 Jun 3;13:871272. doi: 10.3389/fendo.2022.871272 (PMC9203723; doi:10.3389/fendo.2022.871272)
Supplement: Supplementary file 2 [file DataSheet_2.docx]

**Search strategies**

**Pubmed and Cochrane Central Register of Controlled Trials**

#1 diabetic nephropathy.tw

#2 diabetic kidney disease.tw

#3 diabetic glomerulopathy.tw

#4 chronic kidney disease in type 2 diabetes.tw

#5 renal dysfunction in type 2 diabetes.tw

#6 impaired renal function in type 2 diabetes.tw

#7 renal impairment in type 2 diabetes.tw

#8 renal insufficiency in type 2 diabetes.tw

#9 decline in renal function in type 2 diabetes.tw

#10 #1 OR #2 OR #3 OR #4 OR #5 OR #6 OR #7 OR #8 OR #9

#11 non-proteinuria.tw

#12 non proteinuria.tw

#13 silent.tw

#14 without protein.tw

#15 non-albuminuria.tw

#16 non albuminuria.tw

#17 without albuminuria.tw

#18 normoalbuminuria.tw

#19 normoproteinuria.tw

#20 #11 OR #12 OR #13 OR #14 OR #15 OR #16 OR #17 OR #18 OR #19

#21 #10 AND #20

#22 clinical trial.pt

#23 clinical study.pt

#24 #22 OR #23

#25 #21 AND #24

**Embase**

#1 (('diabetic'/exp OR diabetic) AND ('nephropathy'/exp OR nephropathy) OR (('diabetic'/exp OR diabetic) AND ('kidney'/exp OR kidney) AND ('disease'/exp OR disease)) OR (('diabetic'/exp OR diabetic) AND ('glomerulopathy'/exp OR glomerulopathy)) OR (chronic AND ('kidney'/exp OR kidney) AND ('disease'/exp OR disease) AND in AND type AND ('2'/exp OR 2) AND ('diabetes'/exp OR diabetes)) OR (('renal'/exp OR renal) AND dysfunction AND in AND type AND ('2'/exp OR 2) AND ('diabetes'/exp OR diabetes)) OR (impaired AND ('renal'/exp OR renal) AND ('function'/exp OR function) AND in AND type AND ('2'/exp OR 2) AND ('diabetes'/exp OR diabetes)) OR (('renal'/exp OR renal) AND ('impairment'/exp OR impairment) AND in AND type AND ('2'/exp OR 2) AND ('diabetes'/exp OR diabetes)) OR (('renal'/exp OR renal) AND insufficiency AND in AND type AND ('2'/exp OR 2) AND ('diabetes'/exp OR diabetes)) OR (('decline'/exp OR decline) AND ('renal'/exp OR renal) AND ('function'/exp OR function) AND in AND type AND ('2'/exp OR 2) AND ('diabetes'/exp OR diabetes))) AND ('non proteinuria' OR (non AND ('proteinuria'/exp OR proteinuria)) OR silent OR (without AND ('protein'/exp OR protein)) OR 'non albuminuria' OR (non AND ('albuminuria'/exp OR albuminuria)) OR (without AND ('albuminuria'/exp OR albuminuria)) OR 'normoalbuminuria'/exp OR normoalbuminuria OR normoproteinuria)

#2 #1 AND ('clinical study'/de OR 'clinical trial'/de)
